# Variation in Surgical Management of Stage IV Breast Cancer: Local/Institutional and Regional Geographic Considerations

**DOI:** 10.1245/s10434-026-20123-3

**Published:** 2026-07-11

**Authors:** Shruti Zaveri, Zachary A. Whitham, Anvy Nguyen, Arushii Nadar, A. Marilyn Leitch, Glenda Delgado, Deborah Farr, Rachel Wooldridge, Anthony Froix, Stephanie K. Serres

**Affiliations:** 1 Division of Surgical Oncology, Department of Surgery, The University of Texas Southwestern Medical Center, Dallas, TX USA; 2 Division of Hematology/Oncology, Department of Internal Medicine, The University of Texas Southwestern Medical Center, Dallas, TX USA

## Abstract

**Introduction:**

Prior studies have demonstrated variation in surgical management of breast cancer. As randomized controlled trials have increasingly shown a limited survival advantage with surgery in stage IV breast cancer, the present study aimed to assess geographic and institutional variations in locoregional surgical management of de novo stage IV breast cancer.

**Methods:**

We conducted a retrospective cohort analysis of 68,670 patients with de novo stage IV breast cancer from 2012 to 2021 using the National Cancer Database. The proportion of patients treated with surgical resection was compared between year of diagnosis, facility type, geographic region, and facility volume while controlling for demographic and clinical variables.

**Results:**

Overall, 17,620 patients (25.7%) with de novo stage IV breast cancer underwent breast, axillary, or metastatic site surgical resection. There was a statistically significant decrease from 27.4 to 15.0% in the proportion of patients undergoing primary tumor resection over the included timeframe (*p* < 0.001). Significant variation was noted in the proportion of patients receiving primary tumor resection among geographic regions, ranging from 14.9 to 23.2% (Northeast and West, *p* < 0.001). Patients treated at comprehensive community cancer centers were most likely to have primary site surgery, and those treated at academic centers (including National Cancer Institute–designated comprehensive cancer centers) were least likely (22.1 vs. 16.7%, respectively, *p* < 0.001).

**Conclusion:**

Significant variation exists in locoregional surgical management of de novo stage IV breast cancer. Surgical intervention has decreased over time, but this study reveals that institutional and geographic factors are associated with the surgical management for these patients.

Based on Surveillance, Epidemiology, and End Results data, approximately 6% of newly diagnosed breast cancers present with de novo stage IV disease.^1,2^ Historically, the principle guiding management of stage IV breast cancer has been that surgical resection of the primary tumor does not alter the course of metastatic disease. As such, the management of stage IV disease has largely been driven by systemic treatment, with surgical intervention reserved for palliation of local symptoms, such as bleeding and pain, as needed.^3^

Retrospective data have previously been used to assess the benefit of surgical intervention in stage IV disease, and—although these studies have shown mixed results—they have often favored locoregional treatment.^4–7^ However, retrospective studies in patients with stage IV breast cancer are often impacted by selection bias, stage migration bias, and immortal time bias favoring the treatment arm.^4–9^ To address these limitations, multiple contemporary randomized controlled trials have been conducted to assess the benefit of surgical intervention for stage IV breast cancer. Four large trials, the Tata Memorial, ECOG-ACRIN 2108, ABCSG-28 POSITIVE, and JCOG 1017 PRIM-BC trials, all found no improved overall survival with surgical intervention. Only one study, the MF07-01 trial, showed improved overall survival in the surgical group.^10^

Despite the randomized controlled trial data, given the heterogeneity of patients with de novo stage IV breast cancer studied, controversy on surgical management remains. Given the conflicting evidence and study limitations, there remain no specific guidelines against surgery in patients with stage IV breast cancer, and decisions for or against surgery are often made on a case-by-case basis, leaving room for significant variation in care, especially for patients with a low burden of metastatic disease. The aim of the current study was to characterize contemporary variation in the management of de novo stage IV breast cancer at the local/regional level and based on surgical volume, particularly within the timeframe following publication of multiple randomized controlled trials showing negligible survival benefit with surgical intervention.

## Methods

### Data Source

This retrospective cohort study used the National Cancer Database (NCDB) from the American College of Surgeons and the American Cancer Society. The NCDB includes a broad array of demographic and clinical data for the purpose of comparative benchmarking among cancer patients in the United States. Over 70% of new cancer diagnoses within the United States are represented, from over 1500 Commission on Cancer–accredited facilities. Data are abstracted at each hospital using standardized criteria and nationally standardized data coding definitions. The University of Texas Southwestern Review Board deemed that the study was exempt from informed consent requirement; all data were deidentified.

### Study Population

We queried the NCDB for patients aged ≥18 years with de novo stage IV breast cancer diagnosed between 2012 and 2021. All patients had International Classification of Diseases for Oncology, Third Edition (ICD-O-3) site codes C50.0–C50.9, indicating breast primary site, and histology codes 8500, 8520, 8522, 8523, 8424, 8530, and 8575, indicating ductal, lobular, inflammatory, or metaplastic carcinoma. Only patients with American Joint Committee on Cancer clinical stage IV disease were included. We excluded patients with a previous history of cancer and cases where the reporting facility did not make any treatment decisions (class of case code 00). We also excluded those with missing sociodemographic, staging, vital status or follow-up, facility type, or location.

### Exposure and Covariates

To characterize variation in the operative management of stage IV breast cancer, we examined several facility-level variables. The NCDB categorizes facility types as community cancer programs, comprehensive community cancer programs, academic/research programs (including National Cancer Institute–designated comprehensive cancer centers), and Integrated Cancer Network programs. The NCDB facility regions variable was recategorized into Northeast (New England and Middle Atlantic), South (South Atlantic, Easy South Central, West South Central), Midwest (East North Central and West North Central), and West (Mountain and Pacific) regions. Both facility-level variables are suppressed in individuals diagnosed before the age of 40 years, but were able to be imputed by matching facility identification information from older patients. The total number of patients receiving care at each facility during the study period was used to calculate the facility volume, which was then broken down into quartiles. Facilities in the first quartile treated up to 44 patients with stage IV breast cancer, those in the second quartile treated 45–78, those in the third quartile treated 79–132, and those in the fourth quartile treated ≥ 133.

Additional sociodemographic and clinical factors were abstracted from the NCDB. Sociodemographic factors included age at diagnosis, sex, race and ethnicity, travel distance and median household income. Sex was categorized as male or female. Race and ethnicity was characterized as non-Hispanic white (white), non-Hispanic Black (Black), Hispanic, Asian, other, and unknown. Each patient’s zip code at diagnosis was used to determine their median household income based on information abstracted from the American Community Survey and categorized into quartiles. Clinical characteristics included year of diagnosis (2012–2014, 2015–2017, and 2018–2021), Charlson–Deyo comorbidity score, tumor histology and grade, receptor subtype (based on hormone receptor [HR] and human epidermal growth factor receptor 2 [HER2]/neu tumor receptor positivity), clinical T and N stage, metastatic site (bone, brain, liver, lung, distant lymph nodes, and other) and burden (number of metastatic sites at presentation).

### Outcomes

The primary outcome was receipt of any surgical therapy. Surgical variables were broken down by surgical site into types of breast/primary site surgery (none, partial mastectomy, mastectomy, unknown), axillary surgery (none, sentinel lymph node biopsy [SLNB], axillary lymph node dissection [ALND], and Unknown), and surgery of other sites (none, procedure not specified, regional site, distant lymph nodes, distant site, combination of categories, and unknown).

### Statistical Analysis

Patients were grouped by receipt of any surgical therapy. Descriptive statistics were reported using median (interquartile range [IQR]) and frequency (%) for continuous and categorical variables, respectively. Differences in sociodemographic and clinical factors between groups were determined by Wilcoxon Rank Sum and chi-squared tests for continuous and categorical data, respectively. To assess variation in care across facility-level factors and time, proportions of patients receiving systemic therapy alone, systemic therapy + surgery, systemic therapy + radiation, and trimodal therapy were computed. The proportion of patients undergoing primary site surgery across the same factors were calculated and differences in these outcomes were determined using chi-squared tests. Additionally, variation in the type of primary site surgery (partial mastectomy vs. mastectomy) and axillary intervention was assessed. Multivariable logistic regression was used to determine factors associated with primary site surgery. Factors significant on univariable analysis and those of a priori clinical significance were included in the final model. Scatterplots comparing facility volume with proportion of patients receiving primary site surgery stratified by facility type were created to better visualize the variation in care. All statistical analyses were performed using STATA software (StataCorp. 2023. Stata Statistical Software: Release 18. College Station, TX, USA; StataCorp LLC.).

## Results

### Study Cohort

We identified a total of 68,670 patients with de novo stage IV breast cancer diagnosed between 2012 and 2021 in the NCDB who met the inclusion criteria. Over 98% of the study population was female, and the median age of patients overall was 61 years (IQR 51–71, Table 1). Most patients were non-Hispanic white (47,691 [69.5%]), followed by Black (12,190 [17.8%]), Hispanic (4288 [6.2%]), Asian (2650 [3.9%]), and other races (1232 [1.8%]). In terms of tumor characteristics, 85.9% of patients had invasive ductal carcinoma, 11.8% invasive lobular carcinoma, 1.8% inflammatory breast cancer, and 0.5% metaplastic. In total, 53.6% of patients had HR+/HER2- disease, 13.0% had HR+/HER2+ disease, 12.3% had triple-negative disease, and 7.1% had HR-/HER2+ disease. Most patients had poorly (41.0%) or moderately (37.1%) differentiated tumors. In terms of metastatic burden, 56.0% of patients who underwent surgery had one distant disease site, 25.5% had two distant disease sites, 12.5% had three sites, and 5.9% had four or more sites. Most of these patients had bone metastases (70.1%), followed by lung (30.3%), liver (25.5%), distant lymph nodes (24.3%), brain (7.7%), and other sites (11.9%).

**Table 1:** Cohort demographics

Characteristic	Overall (*n* = 68,670)	No surgery (*n* = 51,050)	Any surgery (*n* = 17,620)	*p*-value
Age, years	61 (51–71)	62 (53–72)	58 (48–67)	< 0.001
Age categories, years				
< 45	9937 (14.5)	6338 (12.4)	3599 (20.4)	< 0.001
46–55	13,565 (19.8)	8356 (18.3)	4209 (23.9)	
56–65	19,104 (27.8)	14,278 (28.0)	4826 (27.4)	
66–75	14,825 (21.6)	11,657 (22.8)	3168 (18.0)	
≥ 76	11,239 (16.4)	9421 (18.5)	1818 (10.3)	
Sex, female	67,700 (98.6)	50,399 (98.7)	17,301 (98.2)	< 0.001
Race				
White	47,691 (69.5)	35,359 (69.3)	12,332 (70.0)	< 0.001
Black	12,190 (17.8)	9327 (18.3)	2863 (16.3)	
Hispanic	4288 (6.2)	3044 (6.0)	1244 (7.1)	
Asian	2650 (3.9)	1917 (3.8)	733 (4.2)	
Other	1232 (1.8)	923 (1.8)	309 (1.8)	
Unknown	619 (0.9)	480 (0.9)	139 (0.8)	
Year of diagnosis				
2012–2014	18,419 (26.8)	12,435 (24.4)	5984 (34.0)	< 0.001
2015–2017	20,691 (30.1)	15,396 (30.2)	5295 (30.1)	
2018–2021	29,560 (43.1)	23,219 (45.5)	6341 (36.0)	
Charlson–Deyo comorbidity score				
0	55,758 (81.2)	41,082 (80.5)	14,676 (83.3)	< 0.001
1	8530 (12.4)	6428 (12.6)	2102 (11.9)	
2	2521 (3.7)	1993 (3.9)	528 (3.0)	
3	1861 (2.7)	1547 (3.0)	314 (1.8)	
Histology				
IDC	59,002 (85.9)	43,604 (85.4)	15,398 (87.4)	< 0.001
ILC	8099 (11.8)	6385 (12.5)	1714 (9.7)	
Inflammatory	1210 (1.8)	880 (1.7)	330 (1.9)	
Metaplastic	359 (0.5)	181 (0.4)	178 (1.0)	
Receptor subtype				
Triple negative	8448 (12.3)	5850 (11.5)	2598 (14.7)	< 0.001
HR-/HER2+	4873 (7.1)	3431 (6.7)	1442 (8.2)	
HR+/HER2-	36,808 (53.6)	28,133 (55.1)	8675 (49.2)	
HR+/HER2+	8927 (13.0)	6442 (12.6)	2485 (14.1)	
Unknown	9714 (14.0)	7194 (14.1)	2420 (13.7)	
Grade				
Well differentiated	4254 (6.2)	3406 (6.7)	848 (4.8)	< 0.001
Moderately differentiated	25,463 (37.1)	19,503 (38.2)	5960 (33.8)	
Poorly differentiated	38,156 (41.0)	19,331 (37.9)	8825 (50.1)	
Unknown	10,797 (15.7)	8810 (17.3)	1987 (11.3)	
Clinical T stage				
T1	7268 (10.6)	5244 (10.3)	2024 (11.5)	< 0.001
T2	20,499 (29.9)	14,801 (29.0)	5698 (32.3)	
T3	11,192 (16.3)	8175 (16.0)	3017 (17.1)	
T4	21,919 (31.9)	16,628 (32.6)	5291 (30.0)	
Unknown	7792 (11.4)	6202 (12.2)	1590 (9.0)	
Clinical N stage				
N0	13,561 (19.8)	9898 (19.4)	3663 (20.8)	< 0.001
N1	31,117 (45.3)	23,355 (45.6)	7762 (44.1)	
N2	7668 (11.2)	5575 (10.9)	2093 (11.9)	
N3	9925 (14.5)	7137 (14.0)	2788 (15.8)	
Unknown	6399 (9.3)	5085 (10.0)	1314 (7.5)	
Metastatic site				
Bone	48,104 (70.1)	37,348 (73.2)	10,756 (61.0)	< 0.001
Brain	5256 (7.7)	4248 (8.3)	1008 (5.7)	< 0.001
Liver	17,527 (25.5)	14,125 (27.7)	3402 (19.3)	< 0.001
Lung	20,820 (30.3)	16,592 (32.5)	4228 (24.0)	< 0.001
Distant lymph nodes	16,701 (24.3)	12,488 (24.5)	4213 (23.9)	0.141
Other sites	8160 (11.9)	6625 (13.0)	1535 (8.7)	< 0.001
Metastatic burden				
1 site	38,482 (56.0)	26,052 (51.0)	12,430 (70.5)	< 0.001
2 sites	17,536 (25.5)	14,093 (27.6)	3443 (19.5)	
3 sites	8613 (12.5)	7342 (14.4)	1271 (7.2)	
4 or more sites	4039 (5.9)	3563 (7.0)	476 (2.7)	
Breast surgery type				
None	55,021 (80.1)	51,050 (100)	3971 (22.5)	< 0.001
Partial mastectomy	3769 (5.5)	0 (0)	3769 (21.4)	
Mastectomy	9839 (14.3)	0 (0)	9839 (55.8)	
Unknown	41 (0.1)	0 (0)	41 (0.2)	
Axillary surgery type				
None	56,587 (82.4)	51,050 (100)	5537 (31.4)	< 0.001
SLNB	2641 (3.9)	0 (0)	2641 (15.0)	
ALND	9155 (13.4)	0 (0)	9166 (52.0)	
Unknown	276 (0.4)	0 (0)	276 (1.6)	
Other site surgery				< 0.001
None	64,433 (93.8)	51,050 (100)	13,383 (76.0)	
Procedure not specified	600 (0.9)	0 (0)	600 (3.4)	
Regional site	516 (0.8)	0 (0)	516 (2.9)	
Distant lymph nodes	475 (0.7)	0 (0)	475 (2.7)	
Distant site	2490 (3.6)	0 (0)	2490 (14.1)	
Combination	76 (0.1)	0 (0)	76 (0.4)	
Unknown	80 (0.1)	0 (0)	80 (0.5)	
Systemic therapy				< 0.001
No	5091 (7.4)	4423 (8.7)	668 (3.8)	
Yes	59,830 (87.1)	43,414 (85.0)	16,416 (93.2)	
Unknown	3749 (5.5)	3213 (6.3)	536 (3.0)	
Distance traveled, miles	9.0 (4.2–19.8)	8.7 (4.1–19.1)	9.9 (4.6–21.8)	< 0.001
< 5	17,873 (26.0)	13,782 (27.0)	4091 (23.2)	< 0.001
5–10	14,022 (20.4)	10,528 (20.6)	3494 (19.8)	
10–20	12,847 (18.7)	9429 (18.5)	3148 (19.4)	
> 20	14,792 (21.5)	10,635 (20.8)	4157 (23.6)	
Unknown	9136 (13.3)	6676 (13.1)	2460 (14.0)	

Data are presented as median (interquartile range) or n (%) unless otherwise indicated

*ALND* Axillary lymph node dissection, *HER*2 Human epidermal growth factor receptor 2, *HR* Hormone receptor, *IDC* Invasive ductal carcinoma, *ILC* Invasive lobular carcinoma, *SLNB* Sentinel lymph node biopsy

The majority of patients (51,050 [74.3%]) did not undergo surgery as a part of their stage IV breast cancer treatment, whereas a select minority (17,620 [25.7%]) underwent surgery either for the primary breast and/or axillary lesion or at a metastatic site (Table 1). Patients who underwent surgery were younger (median age 58 years [IQR 48–67] vs. median age 62 years [IQR 53–72], *p* < 0.001), more likely to have HER2+ or triple-negative disease (37.0% vs. 30.8%, *p* < 0.001), and more likely to have a lower metastatic burden (70.5% vs. 50.0% with only one metastatic site, *p* < 0.001). Among the overall cohort, 19.8% of patients underwent breast surgery (partial mastectomy or mastectomy), 17.3% of patients underwent axillary surgery (sentinel lymph node biopsy or axillary lymph node dissection), and 4.4% of patients underwent surgery at a metastatic site (distant disease site or distant lymph node resection). Among those who underwent surgery of any type, 55.8% had a mastectomy, 21.4% had a lumpectomy, and 22.5% had no primary breast surgery (had axillary or metastatic site resection alone). In terms of axillary management in patients who underwent surgery of any type, 52.0% had an ALND, 15.0% had an SLNB, and 31.4% had no axillary surgery. Among patients who received surgery, 24.0% underwent surgical resection of a metastatic site. The vast majority (93.2%) of patients who underwent surgery also received systemic therapy, whereas only 85.0% of the ‘no surgery’ cohort received systemic therapy (*p* < 0.001).

### Variation in Treatment

#### Variation Across Study Timeframe

There was a statistically significant decrease from 27.4 to 15.0% in the proportion of patients undergoing primary-site surgery over the included timeframe (*p* < 0.001, Table 2). When divided across three timeframes (2012–2014, 2015–2017, and 2018–2021), there was a significant decrease in trimodal therapy, from 15.4 to 10.9% (*p* < 0.001), a significant decrease in the use of systemic therapy plus surgery, from 16.4 to 9.7% (*p* < 0.001), and an increase from 42.0 to 50.1% in systemic therapy alone as treatment for patients with de novo stage IV breast cancer (Table 3).

**Table 2:** Variation in primary site surgery by institutional factors

Facility type	Number of patients with metastases seen	Number of patients with metastases undergoing surgery	Proportion of patients with metastases undergoing surgery (%)	*p*-value
Facility type				< 0.001
Community	5520	1200	21.7	
Comprehensive community	26,139	5782	22.1	
Academic/research	23,369	3903	16.7	
Integrated	13,601	2723	20.0	
Facility region				< 0.001
Northeast	14,141	2112	14.9	
South	26,525	5769	21.8	
Midwest	16,695	3113	18.7	
West	11,268	2614	23.2	
Diagnosis year				< 0.001
2012–2014	18,406	5048	27.4	
2015–2017	20,684	4137	20.0	
2018– 2021	29,539	4423	15.0	
Facility volume				< 0.001
First quartile	17,903	3955	22.1	
Second quartile	17,645	3723	21.1	
Third quartile	16,667	3076	18.5	
Fourth quartile	16,414	2854	17.4

**Table 3:** Variation in multidisciplinary management by institutional factors

Variable	No therapy (*n* = 6406)	Systemic therapy alone (*n* = 31,318)	Systemic therapy + surgery (*n* = 8214)	Systemic therapy + radiation (*n* = 12,096)	Trimodal therapy (*n* = 8202)	*p*-value
Year of diagnosis						< 0.001
2012–2014	1608 (9.1)	7433 (42.0)	2896 (16.4)	3049 (17.2)	2718 (15.4)	
2015–2017	1874 (9.4)	8572 (48.0)	2540 (12.7)	3579 (18.0)	3378 (11.9)	
2018-2021	2924 (10.2)	14,313 (50.1)	2778 (9.7)	5468 (19.1)	3106 (10.9)	
Facility type						< 0.001
Community	680 (12.9)	2313 (43.8)	772 (14.6)	973 (18.4)	540 (10.2)	
Comprehensive community center	2594 (10.3)	11,424 (45.4)	3330 (13.2)	4578 (18.2)	3236 (12.9)	
Academic/research	1751 (7.7)	11,463 (50.6)	2576 (11.4)	4103 (18.1)	2781 (12.3)	
Integrated	1381 (10.5)	6118 (46.6)	1536 (11.7)	2442 (18.6)	1645 (12.5)	
Region						< 0.001
Northeast	1368 (10.0)	7030 (51.5)	1387 (10.2)	2505 (18.4)	1357 (9.9)	
South	2580 (10.1)	11,690 (45.9)	3522 (13.8)	4447 (17.5)	3243 (12.7)	
Midwest	1433 (8.9)	7762 (47.9)	1920 (11.9)	3092 (19.1)	1994 (12.3)	
West	1025 (9.4)	4836 (44.3)	1385 (12.7)	2052 (18.8)	1608 (14.7)	
Facility volume						< 0.001
First quartile	2248 (13.2)	7540 (44.1)	2338 (13.7)	2997 (17.5)	1963 (11.5)	
Second quartile	1731 (10.2)	7760 (45.6)	2151 (12.6)	3198 (18.8)	2191 (12.9)	
Third quartile	1443 (9.0)	7709 (47.9)	1839 (11.4)	3077 (19.1)	2029 (12.6)	
Fourth quartile	984 (6.1)	8309 (51.9)	1886 (11.8)	2824 (17.6)	2020 (12.6)	

Data are presented as *n* (%) unless otherwise indicated

### Variation by Geographic Region

Significant variation was noted in the proportion of patients receiving primary-site surgical intervention among geographic regions, ranging from 14.9 to 23.2% (Northeast and West, respectively; Midwest 18.7%, South 21.8%; *p* < 0.001, Table 2). Rates of systemic therapy alone were highest in the Northeast (51.5 vs. 47.9% Midwest, 45.9% South, and 44.3% West; *p* < 0.001), and consequently rates of systemic therapy + surgery were lowest in the Northeast (10.2 vs. 11.9% Midwest, 12.7% West, and 13.8% South; *p* < 0.001). Rates of trimodal therapy were also lowest in the Northeast (9.9 vs. 12.3% in the Midwest, 12.7% in the South, and 14.7% in the West; *p* < 0.001, Table 3).

### Variation by Facility Type

**Fig. 1:**
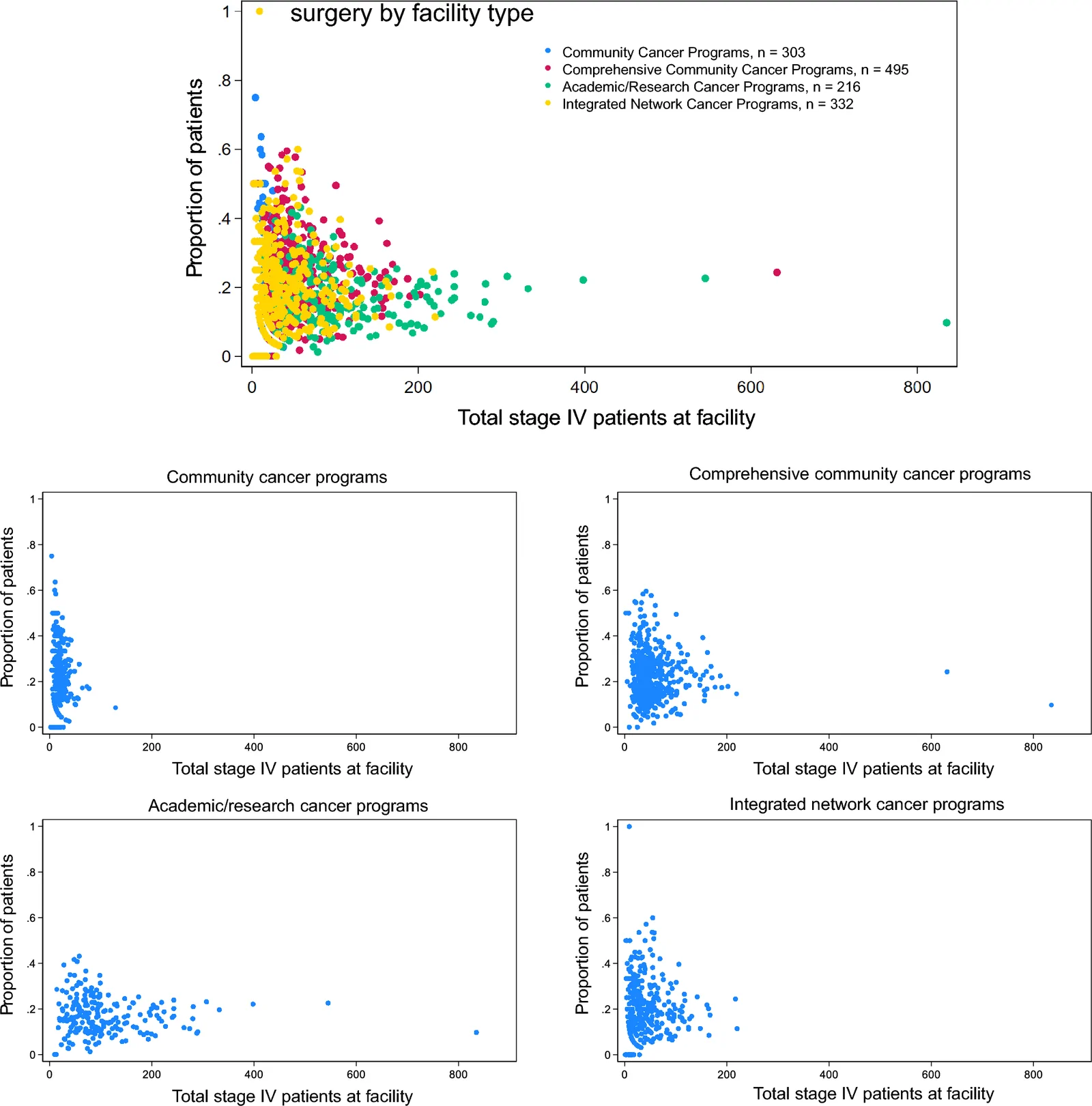
Scatterplots of total volume versus proportion undergoing surgery by facility type

Significant variation in treatment was noted by facility type and volume. Patients treated at comprehensive community centers were most likely to have primary-site surgical intervention, and those treated at academic/research centers (including NCI-designated comprehensive cancer centers) were least likely (22.1 vs. 16.7%%, respectively, *p* < 0.001) (Table 2). Patients treated at community centers had the highest rates of no treatment (surgical, systemic, or radiation), and patients at academic/research centers were least likely to forgo treatment altogether (12.9 vs. 7.7%, *p* < 0.001). Patients treated at community centers also had the lowest rates of systemic therapy alone (44.8 vs. 45.4% at comprehensive cancer centers, 46.6% at integrated cancer centers, 50.6% at academic/research centers, *p* < 0.001). Patients treated at community centers also had the highest rates of systemic therapy + surgery (14.6 vs. 13.2% at comprehensive cancer centers, 11.7% at integrated cancer centers, and 11.4% at academic/research centers, *p* < 0.001) (Table 3). Community centers saw the lowest volumes of patients with stage IV breast cancer but had the highest variation in proportion of patients with stage IV disease receiving surgery (Fig. 1). Conversely, despite seeing a wide range of volumes of patients with stage IV breast cancer, academic centers had consistently lower proportions of those patients receiving surgery (Fig. 1).

### Variation by Facility Volume

Facilities with the lowest volume of patients with stage IV disease were significantly more likely to perform primary-site surgical intervention, with 22.1% undergoing primary-site surgery at facilities in the lowest quartile of stage IV patient volume and 17.4% undergoing primary-site surgery at facilities in the highest quartile of stage IV patient volume (*p* < 0.001, Table 2). Rates of forgoing treatment altogether decreased significantly with stage IV patient volume; facilities seeing the lowest volumes of patients with stage IV breast cancer had the highest rates of no treatment, whereas facilities seeing the highest volumes of patients with stage IV breast cancer had the lowest rates of no treatment (13.2 vs. 6.1%, *p* < 0.001). Rates of systemic therapy alone increased significantly with patient volume; facilities within the first quartile of stage IV patient volume had the lowest rates of systemic therapy alone, whereas facilities within the fourth quartile of patient volume had the highest rates of systemic therapy alone (44.1 vs. 51.9%, *p* < 0.001). Facilities within the first quartile of stage IV patient volume also had the highest rates of systemic therapy + surgery (13.7 vs. 12.6% at second quartile facilities, 11.4% at third quartile facilities, and 11.8% at fourth quartile facilities, *p* < 0.001). The greatest range of variation was seen at the individual facility level, with proportions of patients treated with surgery ranging from 0 to 100% (median 18.8% [IQR 13.1–24.4], Table 3).

### Variation in Type of Primary Site (Breast and/or Axillary) Surgery

Looking more granularly at the type of primary-site surgery, rates of mastectomy decreased over time from 20.1% in 2012–2014 to 10.7% in 2018–2021 but remained higher than rates of lumpectomy in each of the timeframes (7.4 and 4.3%, respectively) (*p* < 0.001). Rates of ALND also decreased over time, from 19.7% in 2012–2014 to 9.3% in 2018–2021 (*p* < 0.001), but were higher than rates of SLNB in each of the timeframes. Rates of mastectomy were highest at comprehensive community centers and lowest at academic centers (16.1 vs. 12.1%, *p* < 0.001). Similarly, rates of ALND were highest at comprehensive community centers and lowest at academic centers (14.6 vs. 12.0%, *p* < 0.001). Rates of mastectomy were also highest at centers within the first quartile of stage IV patient volume and lowest at centers within the fourth quartile (15.7 vs. 12.7%, *p* < 0.001). Similarly, rates of ALND also decreased with increasing stage IV patient volume (14.2% at centers within the first quartile of stage IV patient volume vs. 12.3% at centers within the fourth quartile, *p* < 0.001) (Table 4).

### Factors Associated with Primary Site Surgery

On univariate analysis, patient age, sex, race/ethnicity, year of diagnosis, income quartile, Charlson–Deyo comorbidity score, histology, receptor status, grade, metastatic site and burden, geographic region, facility type and volume, and distance traveled were all factors significantly associated with primary site surgery. On multivariate analysis, not only did the patient demographic and clinical variables remain significant, but also the facility-related variables, such as geographic region, facility type, facility volume, and distance traveled also remained significantly associated with primary site surgery. Patients treated at facilities in the Northeast were least likely to receive primary site surgery (compared with odds ratio [OR] 1.27 [95% confidence interval (CI) 1.18–1.36] in the Midwest; OR 1.55 [95% CI 1.46–1.66] in the South; and OR 1.62 [95% CI 1.50–1.74] in the West). Academic/research centers had lower odds of primary site surgery than did community centers (OR 0.79 [95% CI 0.71–0.87]), and the odds of primary site surgery decreased significantly with increasing patient volume (Table 3). Greater distance traveled to the treatment facility was also associated with significantly increased odds of primary site surgery (> 20miles traveled, OR 1.23 [95% CI 1.16–1.31]) (Table 5).

**Table 4:** Variation in type of primary site surgery

Variable	Breast surgery				Axillary surgery			
	None (*n* = 55,062)	Partial mastectomy (*n* = 3769)	Mastectomy (*n* = 9839)	*p*-Value	None (*n* = 56,863)	SLNB (*n* = 2641)	ALND (*n* = 9166)	*p*-Value
Year of diagnosis								
2012–2014	13,371 (72.6)	1354 (7.4)	3694 (20.1)	< 0.001	14,189 (77.0)	607 (3.3)	3623 (19.7)	< 0.001
2015–2017	16,554 (80.0)	1154 (5.6)	2983 (14.4)		17,171 (83.0)	730 (3.5)	2790 (13.5)	
2018–2021	25,137 (85.0)	1261 (4.3)	3162 (10.7)		25,503 (86.3)	1304 (4.4)	2753 (9.3)	
Facility type								
Community	4327 (78.3)	332 (6.0)	868 (15.7)	< 0.001	4541 (82.2)	208 (3.8)	778 (14.1)	< 0.001
Comprehensive community center	20,363 (77.9)	1583 (6.1)	4199 (16.1)		21,212 (81.1)	1110 (4.3)	3823 (14.6)	
Academic/research	19,485 (83.3)	1075 (4.6)	2828 (12.1)		19,812 (84.7)	769 (3.3)	2807 (12.0)	
Integrated	10,887 (80.0)	779 (5.7)	1944 (14.3)		11,298 (83.0)	554 (4.1)	1758 (12.9)	
Region								
Northeast	12,033 (85.1)	709 (5.0)	1403 (9.9)		12,326 (87.1)	387 (2.7)	1432 (10.1)	
South	20,780 (78.3)	1453 (5.5)	4316 (16.3)		21,539 (81.1)	1064 (4.0)	3946 (14.9)	
Midwest	13,588 (81.4)	836 (5.0)	2277 (13.6)		13,962 (83.6)	666 (4.0)	2073 (12.4)	
West	8661 (76.8)	771 (6.8)	1843 (16.4)		9036 (80.1)	524 (4.7)	1715 (15.2)	
Facility volume								
First quartile	13,960 (77.9)	1112 (6.2)	2843 (15.7)	< 0.001	14,662 (81.8)	714 (4.0)	2539 (14.2)	< 0.001
Second quartile	13,931 (78.9)	1041 (5.9)	2682 (15.2)		14,469 (82.0)	680 (3.9)	2505 (14.2)	
Third quartile	13,600 (81.6)	842 (5.1)	2234 (13.4)		13,951 (83.7)	621 (3.7)	2104 (12.6)	
Fourth quartile	13,571 (82.6)	774 (4.7)	2080 (12.7)		13,781 (83.9)	626 (3.8)	2018 (12.3)	

Data are presented as n (%) unless otherwise indicated.

*ALND* Axillary lymph node dissection, *SLNB* Sentinel lymph node biopsy

**Table 5:** Factors associated with primary site surgery

Factor	Univariate		Multivariate	
	OR (95% CI)	*p*-Value	OR (95% CI)	*p*-Value
Age category, years				
< 45	Ref	Ref	Ref	Ref
46–55	0.74	0.70–0.79	0.72	0.67–0.77
56–65	0.54	0.51–0.58	0.56	0.53–0.60
66–75	0.44	0.42–0.47	0.46	0.43–0.49
≥ 76	0.33	0.30–0.35	0.32	0.29–0.34
Sex				
Female	Ref	Ref	Ref	Ref
Male	1.44	1.25–1.67	1.73	1.46–2.04
Race and ethnicity				
White	Ref	Ref	Ref	Ref
Black	0.87	0.83–0.92	0.79	0.74–0.84
Hispanic	1.22	1.13–1.31	1.11	1.02–1.22
Asian	1.15	1.05–1.27	0.96	0.86–1.08
Other	0.97	0.83–1.12	0.91	0.77–1.08
Unknown	0.78	0.63–0.97	0.75	0.59–0.96
Income quartiles				
First	Ref	Ref	Ref	Ref
Second	1.12	1.03–1.21	1.07	0.98–1.17
Third	1.09	1.02–1.18	1.08	0.99–1.17
Fourth	1.10	1.02–1.18	1.12	1.03–1.21
Year of diagnosis				
2012–2014	Ref	Ref	Ref	Ref
2015–2017	0.66	0.63–0.69	0.68	0.63–0.73
2018–2021	0.47	0.45–0.49	0.49	0.45–0.54
Charlson–Deyo comorbidity score				
0	Ref	Ref	Ref	Ref
1	0.91	0.86–0.97	0.98	0.91–1.05
2	0.67	0.60–0.75	0.85	0.74–0.96
3	0.49	0.73–0.57	0.70	0.59–0.83
Histology				
IDC	Ref	Ref	Ref	Ref
ILC	0.61	0.57–0.65	0.85	0.78–0.92
Inflammatory	1.14	1.00–1.31	1.02	0.87–1.20
Metaplastic	3.51	2.85–4.33	3.36	2.61–4.31
Receptor subtype				
Triple negative	Ref	Ref	Ref	Ref
HR-/HER2+	0.96	0.88–1.04	1.07	0.97–1.18
HR+/HER2-	0.64	0.60–0.67	0.84	0.78–0.90
HR+/HER2+	0.86	0.80–0.93	0.98	0.90–1.07
Unknown	0.68	0.63–0.73	0.76	0.69–0.82
Grade				
Well differentiated	Ref	Ref	Ref	Ref
Moderately differentiated	1.28	1.17–1.41	1.33	1.20–1.47
Poorly differentiated	2.09	1.91–2.29	2.04	1.83–2.26
Unknown	0.59	0.53–0.65	0.68	0.60–0.77
Clinical T stage				
T1	Ref	Ref	Ref	Ref
T2	1.09	1.02–1.16	1.05	0.97–1.13
T3	1.08	1.01–1.16	1.02	0.93–1.11
T4	0.96	0.90–1.03	0.99	0.92–1.08
Unknown	0.48	0.44–0.53	0.65	0.58–0.73
Clinical N stage				
N0	Ref	Ref	Ref	Ref
N1	0.82	0.78–0.86	0.75	0.71–0.80
N2	1.01	0.95–1.09	0.93	0.86–1.01
N3	1.05	0.99–1.12	0.88	0.81–0.95
Unknown	0.58	0.53–0.63	0.82	0.74–0.91
Metastatic site				
Bone	0.53	0.51–0.55	0.74	0.83–1.03
Brain	0.38	0.34–0.41	0.63	0.45–0.88
Liver	0.64	0.61–0.67	0.78	0.56–1.08
Lung	0.64	0.61–0.67	0.93	0.67–1.30
Distant lymph nodes	0.92	0.88–0.96	1.38	0.99–1.92
Other sites	0.54	0.50–0.58	0.82	0.32–0.62
Metastatic burden				
1 site	Ref	Ref	Ref	Ref
2 sites	0.43	0.41–0.45	0.44	0.32–0.62
3 sites	0.27	0.25–0.29	0.30	0.15–0.58
≥4 sites	0.17	0.15–0.19	0.24	0.08–0.70
Facility type				
Community	Ref	Ref	Ref	Ref
Comprehensive community	1.02	0.95–1.10	1.07	0.98–1.17
Academic/research	0.72	0.67–0.78	0.79	0.71–0.87
Integrated	0.90	0.83–0.97	0.93	0.85–1.03
Facility region				
Northeast	Ref	Ref	Ref	Ref
South	1.58	1.50–1.67	1.55	1.46–1.66
Midwest	1.31	1.23–1.39	1.27	1.18–1.36
West	1.72	1.61–1.83	1.62	1.50–1.74
Distance traveled, miles				
< 5	Ref	Ref	Ref	Ref
5–10	1.12	1.06–1.19	1.08	1.02–1.15
10–20	1.22	1.15–1.29	1.14	1.07–1.21
> 20	1.24	1.18–1.31	1.23	1.16–1.31
Unknown	1.16	1.09–1.24	5.80	0.35–96.00
Facility volume				
First quartile	Ref	Ref	Ref	Ref
Second quartile	0.94	0.90–0.99	0.89	0.84–0.95
Third quartile	0.80	0.76–0.84	0.77	0.72–0.83
Fourth quartile	0.74	0.70–0.78	0.70	0.65–0.76

*CI* Confidence interval, *HER*2 Human epidermal growth factor receptor 2, *HR* Hormone receptor, *IDC* Invasive ductal carcinoma, *ILC* Invasive lobular carcinoma, *OR* Odds ratio, *ref* reference.

## Discussion

In this large-scale, nationally representative retrospective cohort study, we found significant variation in locoregional management of patients with de novo stage IV breast cancer. The paradigm for management of stage IV breast cancer has predominantly been treatment with systemic therapy alone, with surgery reserved only for palliation of symptoms under the guiding principle that primary-site locoregional surgery would not alter the course of metastatic disease. In recent years, several retrospective studies and five randomized controlled trials have re-examined this paradigm, with four of them (the Tata Memorial trial in India, the Austrian POSYTIVE trial, E2108, JCOG 1017 PRIM-BC) showing no improved outcomes with primary-site surgery.^11–14^ Despite conflicting evidence on the role of locoregional therapy in patients with an intact breast primary, studies show an 18–44% rate of surgery in patients with de novo stage IV breast cancer.^3,8,15^

In this study, we found that 25.7% of patients with de novo stage IV breast cancer underwent surgery at centers captured within the NCDB between 2012 and 2021; 19.8% of patients underwent breast surgery, 17.3% of patients underwent axillary surgery, and 4.4% of patients underwent surgery at a metastatic site. As noted in previous studies, patients with stage IV disease who underwent surgical resection were younger, more likely to have HER2+ or triple-negative disease, and more likely to have a lower burden of metastatic disease, but this study showed significant variation in the surgical management of de novo stage IV breast cancer by facility-level factors such as geographic region and facility type. With regard to geography, the Northeast had the highest rates of systemic therapy alone and the lowest rates of primary site surgery, whereas the West had lower rates of systemic therapy and the highest rates of primary site surgery. With regard to variation by facility type, comprehensive community cancer centers had the highest rates of primary site surgery, whereas academic centers had the lowest rates. There may be interaction between the variation in surgical patterns based on geography and facility type, potentially driven by varying concentrations of academic versus community centers in each geographic region; however, both factors remained significantly associated with surgery in multivariate analyses. Most importantly, patients receiving treatment at centers with lower volumes of stage IV breast cancer were far more likely to undergo primary tumor resection despite having distant metastatic disease, and—among those patients with stage IV disease receiving surgical intervention—centers with lower volumes are more likely to recommend mastectomy and an ALND.

Variation has previously been noted in the surgical management of breast cancer, with differences in management noted based on a range of factors, including patient demographic and clinical factors, and surgeon, institutional, and geographic factors.^16–19^ Importantly, institution-level factors, such as availability of plastic surgery, have been shown to account for a significant amount of surgical variation. Specific to management of stage IV breast cancer, variation in surgical management was previously considered using the NCDB from 2003 to 2012, where 44% of patients underwent surgery, with a very slight decrease in surgical intervention noted during the study timeframe.^3^ More recently, in a study considering the years 2013–2016, 24% of patients underwent surgical resection.^9^ Our study does show a further decrease in the rates of primary site surgery in stage IV breast cancer, but this decrease is not seen uniformly across institutions.

In an era of novel systemic therapies and improved durable response to systemic therapy, the role of primary-site surgery for stage IV breast cancer is still evolving. In patients with oligometastatic disease or a very limited burden of metastatic disease, surgical resection of the primary tumor is sometimes recommended by the multidisciplinary team as a part of curative-intent treatment. Our data show that more patients who underwent surgery also received systemic therapy (93.2%), whereas only 85.0% of the ‘no surgery’ cohort received systemic therapy (*p* < 0.001), probably because patients undergoing surgery were doing so with a plan for curative-intent treatment. If surgical intervention is to be pursued, it must be done in the context of complete evaluation of the metastatic burden and with multidisciplinary consensus. The finding of higher rates of surgery at centers with lower stage IV patient volumes raises concern for potential mismanagement or may indicate patient-level factors driving these decisions. Institutions seeing lower volumes of patients with stage IV disease may perform surgery in cases where institutions with greater experience with stage IV breast cancer may deem surgery futile. Recognizing these differences in practice patterns between institutions raises awareness of the need for further standardization of care.

The findings of this study must be considered within the context of its limitations. Although the NCDB is subject to rigorous quality assurance measures, the data are collected retrospectively and are susceptible to potential confounding variables. Given the retrospective nature of the study, our data can only demonstrate an association between the geographic or institutional factors and surgical management rather than infer causality. Furthermore, errors in the documentation and data procurement are possible. The data do not allow for the granularity required to assess specific reasons for surgical intervention in any given case that could impact the surgical decision-making. The reason for surgery (palliative vs. curative intent) cannot be determined. The data also cannot be used to suggest a reason for the variation in surgical patterns. For instance, we cannot ascertain whether surgery was more frequently offered by providers or requested by patients at lower-volume centers. The NCDB only includes data from participating Commission on Cancer–accredited institutions. As such, this study only includes hospitals that participate in reporting to the NCDB and does not fully capture the extent of primary site surgery in stage IV breast cancer. True rates of primary site surgery and variation in care may be higher at non-reporting centers.

## Conclusion

This retrospective cohort analysis of patients with de novo stage IV breast cancer shows that significant variation exists in locoregional surgical management of de novo stage IV breast cancer. Surgical intervention has decreased over time, likely because evidence has demonstrated limited survival advantage and improving systemic therapy. However, this study reveals that institutional and geographic factors are significantly associated with variations in the surgical management for these patients. Initiatives to standardize breast cancer care for patients with de novo stage IV disease will need to address specific local and regional factors for variation.

## References

[1] O’Shaughnessy J. Extending survival with chemotherapy in metastatic breast cancer. *Oncologist*. 2005;10(S3):20–9. 16368868 10.1634/theoncologist.10-90003-20

[2] Surveillance. Epidemiology aERSPwscgSSDISRP-U, Nov 2004 Sub (1973–2002), National Cancer Institute, DCCPS, Surveillance Research Program, Cancer Statistics Branch, released April 2005, based on the November 2004 submission. Surveillance, Epidemiology, and End Results Program

[3] Lane WO, Thomas SM, Blitzblau RC, et al. Surgical resection of the primary tumor in women with de novo stage IV breast cancer: contemporary practice patterns and survival analysis. *Ann Surg*. 2019;269(3):537–44. 29227346 10.1097/SLA.0000000000002621 PMC5994388

[4] Gera R, Chehade HELH, Wazir U, Tayeh S, Kasem A, Mokbel K. Locoregional therapy of the primary tumour in de novo stage IV breast cancer in 216 066 patients: a meta-analysis. *Sci Rep*. 2020;10(1):2952. 32076063 10.1038/s41598-020-59908-1 PMC7031518

[5] Gnerlich J, Jeffe DB, Deshpande AD, Beers C, Zander C, Margenthaler JA. Surgical removal of the primary tumor increases overall survival in patients with metastatic breast cancer: analysis of the 1988–2003 SEER data. *Ann Surg Oncol*. 2007;14(8):2187–94. 17522944 10.1245/s10434-007-9438-0

[6] Lang JE, Tereffe W, Mitchell MP, et al. Primary tumor extirpation in breast cancer patients who present with stage IV disease is associated with improved survival. *Ann Surg Oncol*. 2013;20(6):1893–9. 23306905 10.1245/s10434-012-2844-y PMC4353581

[7] Vohra NA, Brinkley J, Kachare S, Muzaffar M. Primary tumor resection in metastatic breast cancer: a propensity-matched analysis, 1988–2011 SEER data base. *Breast J*. 2018;24(4):549–54. 29498453 10.1111/tbj.13005

[8] Arciero C, Liu Y, Gillespie T, Subhedar P. Surgery and survival in patients with stage IV breast cancer. *Breast J*. 2019;25(4):644–53. 31087448 10.1111/tbj.13296 PMC6612438

[9] Duchesneau ED, Jackson BE, Webster-Clark M, et al. The timing, the treatment, the question: comparison of epidemiologic approaches to minimize immortal time bias in real-world data using a surgical oncology example. *Cancer Epidemiol Biomarkers Prev*. 2022;31(11):2079–86. 35984990 10.1158/1055-9965.EPI-22-0495 PMC9627261

[10] Soran A, Ozmen V, Ozbas S, et al. Primary surgery with systemic therapy in patients with de novo stage IV breast cancer: 10-year follow-up; protocol MF07-01 randomized clinical trial. *J Am Coll Surg*. 2021;233(6):742-751.e745. 34530124 10.1016/j.jamcollsurg.2021.08.686

[11] Badwe R, Hawaldar R, Nair N, et al. Locoregional treatment versus no treatment of the primary tumour in metastatic breast cancer: an open-label randomised controlled trial. *Lancet Oncol*. 2015;16(13):1380–8. 26363985 10.1016/S1470-2045(15)00135-7

[12] Fitzal F, Bjelic-Radisic V, Knauer M, et al. Impact of breast surgery in primary metastasized breast cancer: outcomes of the prospective randomized phase III ABCSG-28 POSYTIVE trial. *Ann Surg*. 2019;269(6):1163–9. 31082916 10.1097/SLA.0000000000002771

[13] Khan SA, Zhao F, Solin LJ, et al. A randomized phase III trial of systemic therapy plus early local therapy versus systemic therapy alone in women with de novo stage IV breast cancer: a trial of the ECOG-ACRIN research group (E2108). *J Clin Oncol*. 2020;38(18_suppl):LBA2–LBA2.

[14] Shien T, Hara F, Aogi K, et al. Primary tumour resection plus systemic therapy versus systemic therapy alone in metastatic breast cancer (JCOG1017, PRIM-BC): a randomised clinical trial. *Br J Cancer*. 2025;133(5):625–32. 40615712 10.1038/s41416-025-03097-z PMC12405516

[15] Teshome M. Role of operative management in stage IV breast cancer. *Surg Clin North Am*. 2018;98(4):859–68. 30005779 10.1016/j.suc.2018.03.012

[16] Katz SJ, Lantz PM, Janz NK, et al. Patient involvement in surgery treatment decisions for breast cancer. *J Clin Oncol*. 2005;23(24):5526–33. 16110013 10.1200/JCO.2005.06.217

[17] Hawley ST, Hofer TP, Janz NK, et al. Correlates of between-surgeon variation in breast cancer treatments. *Med Care*. 2006;44(7):609–16. 16799355 10.1097/01.mlr.0000215893.01968.f1

[18] Chiu AS, Thomas P, Killelea BK, Horowitz N, Chagpar AB, Lannin DR. Regional variation in breast cancer surgery: results from the national cancer database (NCDB). *Am J Surg*. 2017;214(5):907–13. 28736057 10.1016/j.amjsurg.2017.07.008

[19] Dankwa-Mullan I, George J, Roebuck MC, et al. Variations in breast cancer surgical treatment and timing: determinants and disparities. *Breast Cancer Res Treat*. 2021;188(1):259–72. 33689057 10.1007/s10549-021-06155-1 PMC8233284

